# A CRISPR Interference of CBP and p300 Selectively Induced Synthetic Lethality in Bladder Cancer Cells *In Vitro*: Erratum

**DOI:** 10.7150/ijbs.91212

**Published:** 2024-01-21

**Authors:** Jianfa Li, ChenChen Huang, Tiefu Xiong, Changshui Zhuang, Chengle Zhuang, Yawen Li, Jing Ye, Yaoting Gui

**Affiliations:** 1 Guangdong and Shenzhen Key Laboratory of Male Reproductive Medicine and Genetics, Institute of Urology, Peking University Shenzhen Hospital, Shenzhen-Peking University-the Hong Kong University of Science and Technology Medical Center, Shenzhen 518000, People's Republic of China; 2 Anhui Medical University, Hefei 230000, Anhui Province, People's Republic of China

After publication of our article, it has been found that some errors of inter-duplication in Figure 4 and Figure 5. As shown in Figure 4, the band of “CBP” in 5637 cells in Figure 4G was identical to that in T24 cells in Figure 4H. After checking the original data, the authors found that the pictures of 5637 and T24 cells were saved in the same file. When we disposed the pictures in Photoshop, the picture of 5637 cell was mislabeled as T24 cell. It has been also found that some errors of inter-duplication in Figure 5. As shown in Figure 5D, the EDU assay image of “hTERT-gCBP(-), hUPII-gp300(-)”contained an inter-duplication with “hTERT-gCBP(+), hUPII-gp300(-)”. As shown in Figure 5H, the EDU assay image of “hTERT-gCBP(-), hUPII-gp300(-)”contained an inter-duplication with “hTERT-gCBP(-), hUPII-gp300(+)”. These mistakes were caused by the misplacement of the list of the “EDU assay” images in Figure 5, the poor folder management and incaution of compose type.

The authors apologize for these errors and state that these do not change the scientific conclusions of the article. The correct figures are shown as below.

## Figures and Tables

**Figure 4H:**
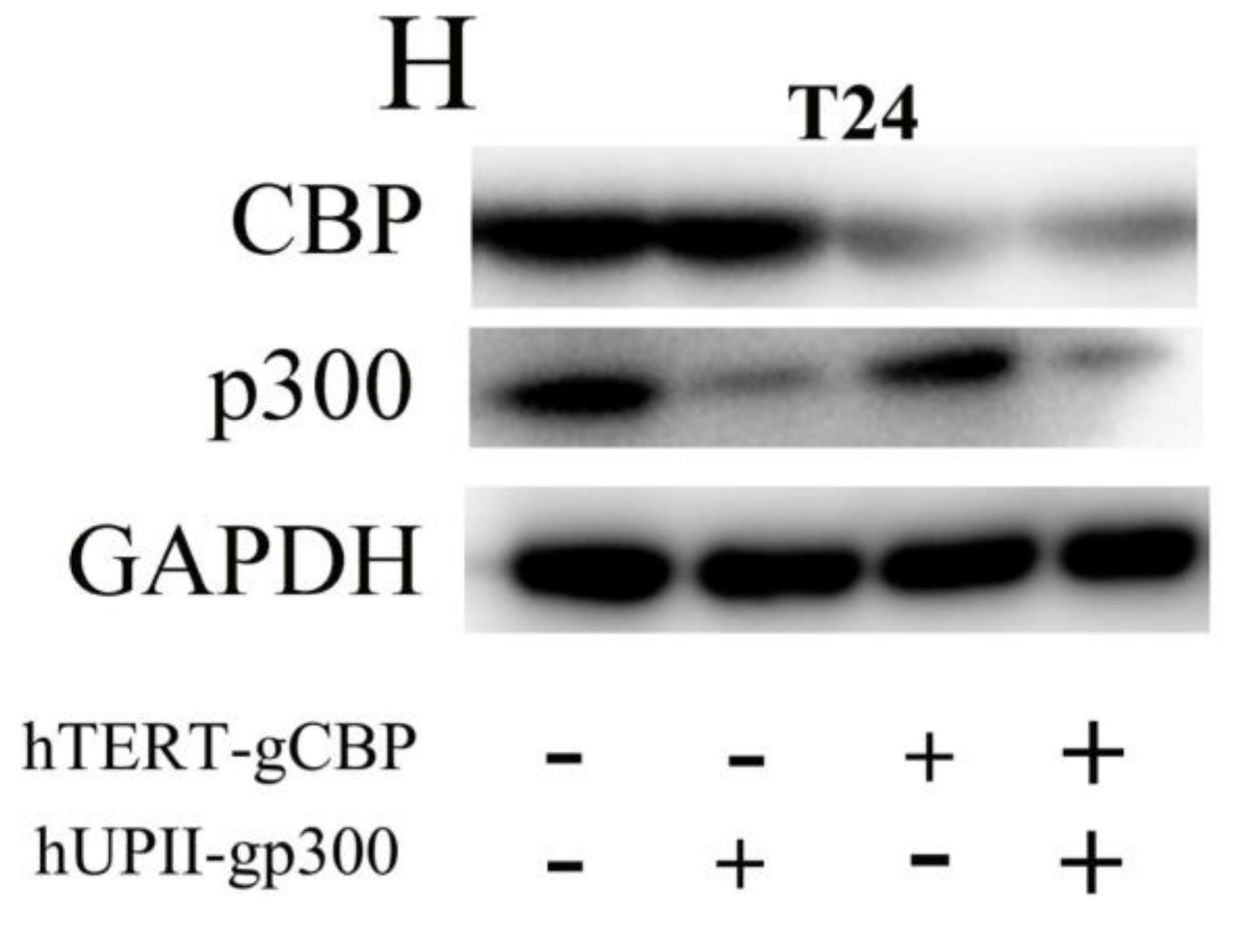
Corrected image.

**Figure 5:**
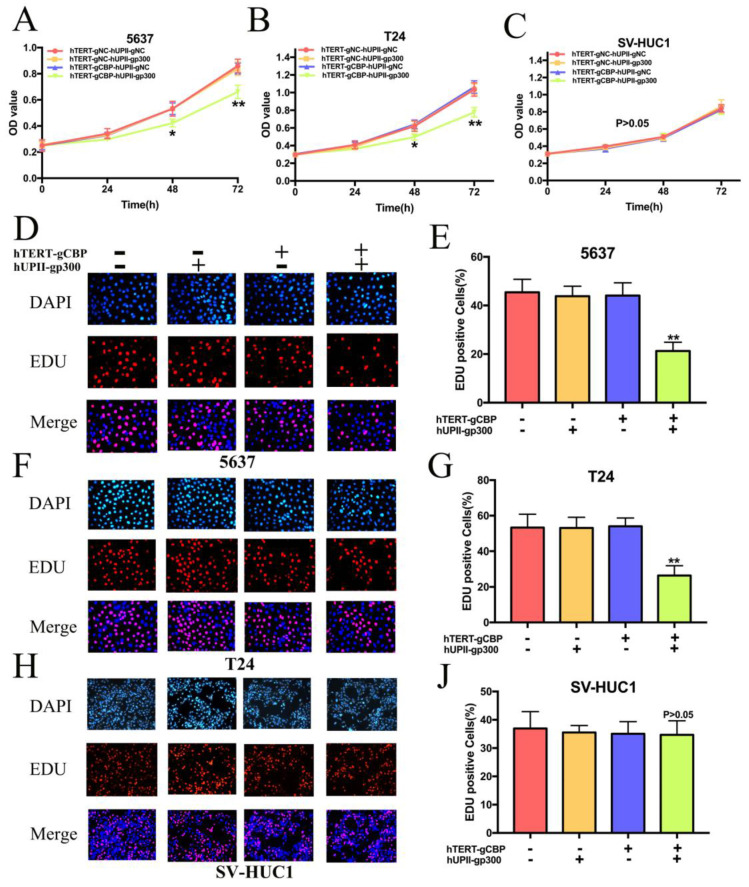
Corrected image.

